# Grape Consumption Increases Anti-Inflammatory Markers and Upregulates Peripheral Nitric Oxide Synthase in the Absence of Dyslipidemias in Men with Metabolic Syndrome

**DOI:** 10.3390/nu4121945

**Published:** 2012-12-06

**Authors:** Jacqueline Barona, Christopher N. Blesso, Catherine J. Andersen, Youngki Park, Jiyoung Lee, Maria Luz Fernandez

**Affiliations:** 1 Department of Nutritional Sciences, University of Connecticut, Storrs, CT 06269, USA; E-Mails: jacqbar@yahoo.com (J.B.); cblesso@gmail.com (C.N.B.); catherine.andersen@uconn.edu (C.J.A.); young-ki.park@uconn.edu (Y.P.); ji-young.lee@uconn.edu (J.L.); 2 School of Microbiology, University of Antioquia, Medellin, A.A. 1226, Colombia

**Keywords:** metabolic syndrome, dyslipidemia, grape polyphenols, IL-10, iNOS

## Abstract

We evaluated the effects of grape consumption on inflammation and oxidation in the presence or absence of dyslipidemias in metabolic syndrome (MetS). Men with MetS (*n* = 24), 11 with high triglycerides and low HDL and 13 with no dyslipidemia were recruited and randomly allocated to consume daily either 46 g of lyophilized grape powder (GRAPE), equivalent to 252 g fresh grapes, or placebo with an identical macronutrient composition and caloric value as GRAPE for four weeks. After a three-week washout, participants followed the alternate treatment. We measured changes between placebo and GRAPE periods in inflammatory and oxidative stress markers both in circulation and in gene expression. Changes in plasma adiponectin (*p* < 0.05), interleukin (IL)-10 (*p* < 0.005) and in mRNA expression of the inducible isoform of nitric oxide synthase (iNOS) (*p* < 0.25) were increased in the GRAPE compared to the placebo period only in those individuals without dyslipidemia. Additionally, plasma IL-10 was negatively correlated with NOX2 expression, a marker of oxidative stress (*r* = −0.55, *p* < 0.01), while iNOS expression was positively correlated with the expression of superoxide dismutase 2 (*r* = 0.642, *p* < 0.01), a key anti-oxidative enzyme. Grape consumption displayed anti-oxidative and increased anti-inflammatory markers in the absence of the inflammatory milieu associated with dyslipidemias.

## 1. Introduction

Metabolic syndrome (MetS) is a group of risk factors associated with insulin resistance that predisposes individuals for type 2 diabetes and cardiovascular disease (CVD), including high blood pressure (BP), dyslipidemia (high triglycerides (TG), low HDL cholesterol (HDL-C)), elevated fasting glucose and abdominal obesity [1,2]. Additionally, people with MetS are characterized by increased oxidative stress [3], a pro-inflammatory state [4,5] and vascular endothelial dysfunction [6]. 

Under conditions of excess adipose tissue, local activation of the NADPH oxidase (NOX) pathway and impaired antioxidant defense systems can increase oxidative stress (*i.e.*, reactive oxygen species (ROS) accumulation) and dysregulate the production of adipokines [3]. Furthermore, this local production of ROS and adipokines can extend to the blood and modulate functions of other tissues [3]. A simultaneous increase in pro-inflammatory cytokines and a decrease in anti-inflammatory cytokines have been observed in people with MetS compared to those without the syndrome [7]. Thus, an imbalance between pro- and anti-inflammatory molecules is involved in the pathogenesis of MetS [3]. For example, dyslipidemic subjects have lower levels of anti-inflammatory HDL and an increased number of pro-inflammatory/atherogenic TG-rich lipoproteins (TGRL) and small, dense LDL [8]. Moreover, pro-inflammatory cytokines induce hypertriglyceridemia and insulin resistance [9], which are main components of MetS. In contrast, reduced levels of the anti-inflammatory IL-10 and adiponectin have been reported in this population [7,10] . Furthermore, low IL-10 production capacity of whole blood has been associated with high plasma glucose and dyslipidemia [11], which would predispose individuals to the MetS and type 2 diabetes, while the opposite situation would confer protection [11].

Polyphenols, which are abundant in fruits and vegetables, have shown antioxidant, anti-inflammatory and hypolipidemic effects [12]. Moreover, *in vitro* studies reviewed in [13,14] have shown that polyphenols inhibit the expression and secretion of pro-inflammatory molecules in several cell lines. This inhibitory action has been shown to be coupled to the enhancement of IL-10 production, thus maintaining the inflammatory:anti-inflammatory ratio [13,14]. Grapes contain numerous polyphenols, such as flavans, anthocyanins, flavonols and stilbenes (e.g., resveratrol), shown to prevent LDL oxidation, oxidative stress, dyslipidemia and inflammation [12,15,16]. These effects may potentially ameliorate MetS and CVD risk factors.

Flow mediated vasodilation (FMD) is used to assess endothelial function, which is mainly dependent on nitric oxide (NO) bioavailability. A reduced FMD has been reported in people with MetS [6]. We have already demonstrated that grape polyphenols increased FMD and reduced systolic blood pressure in the participants of this study [17]. However, the simultaneous evaluation of the effects of grape polyphenols on plasma and mRNA expression of inflammation, oxidative stress markers and peripheral sources of NO, has not been tested in people with MetS—especially those with dyslipidemia that have greater inflammation and/or oxidative stress. Therefore, we aimed (1) to determine the effects of a freeze-dried, whole grape powder, which naturally contains polyphenols, compared with placebo, on plasma pro- and anti-inflammatory and oxidative stress markers in men with MetS based on dyslipidemia classification; and (2) to evaluate the expression of genes related with inflammation, anti-/pro-oxidant enzymes and NO production in peripheral blood mononuclear cells (PBMC) isolated from these individuals.

## 2. Experimental Section

### 2.1. Study Protocol and Population

Twenty four men (30–70 years) classified with MetS according to the revised American Heart Association-National Cholesterol Education Program-Adult Treatment Panel III definition [2] participated in this study. We calculated the sample size based on our previous study with pre- and post-menopausal women in which only 20 individuals were needed to find differences in inflammatory markers and oxidative stress [16]. As previously reported [17], exclusion criteria included renal disease, heart disease, diagnosed diabetes or fasting plasma glucose >126 mg/dL, TG >500 mg/dL, smoking or anti-inflammatory drug use. This study was conducted according to the guidelines laid down in the Declaration of Helsinki, and all procedures involving human subjects were approved by The University of Connecticut-Storrs Institutional Review Board. Written informed consent was obtained from all subjects.

Volunteers were randomly and double-blind assigned to daily consumption of 46 g of a freeze-dried whole grape powder (GRAPE) (equivalent to 2 cups or 252 g of grapes) or to 46 g of placebo in a crossover study. The placebo matched the macronutrient composition (90.1% carbohydrate, 0.6% lipid and 3.2% protein), the energy (1587 kJ/100 g) and physical characteristics of the grape powder, except for the polyphenols. The polyphenol and the nutrient information of GRAPE are reported elsewhere [17]. Briefly, the grape powder contained per 100 g/580 mg of total phenols, 410 mg of flavans and 77 mg of antocyanins. Regarding flavonols, there were 3.1 mg of quercetin, 0.26 mg of myricetin and 0.32 mg of kaempferol per 100 g of lyophilized power. The powder also contained 0.16 mg of resveratrol/100 mg. After four weeks of consuming the GRAPE or placebo supplements, subjects went through a three-week washout period (this amount of time was sufficient to evaluate GRAPE effects on blood pressure and flow-mediated vasodilation [17]) and were assigned to the alternative treatment for an additional four weeks. Participants were asked to avoid consuming polyphenol-rich foods, including grapes, berries and dark chocolate or beverages, including tea and wine, during the whole study. Compliance was assessed using a short questionnaire every week. When compliance was less than 70%, subjects were asked to leave the study. Diet and physical activity were also monitored to ensure no changes occurred in these variables throughout the study. Participants provided a five-day dietary record (including two weekend days) and a seven-day physical activity diary at the baseline and end of each period. 

### 2.2. Classification of Participants

Since dyslipidemic subjects are characterized by a more inflammatory milieu, we decided to separate our participants into two groups to evaluate the effects of grape consumption on two different MetS subsets. Therefore, participants were classified into those who presented both low HDL-C (<40 mg/dL) and high TG (≥150 mg/dL) (dyslipidemic subjects) and those who did not (non-dyslipidemic subjects) at baseline.

### 2.3. Antropometrics, Blood Pressure and Blood Collection

Body weight, height, body mass index (BMI) and waist circumference (WC) were measured as previously reported [17]. Blood was obtained from an antecubital vein using EDTA-coated collection tubes after 12 h overnight fasting. Blood was immediately centrifuged at 2200× *g* for 20 min at 4 °C to separate plasma. A preservation cocktail (1 mL/L sodium azide, 1 mL/L phenylmethylsulphonyl fluoride and 5 mL/L aprotinin) was added to the plasma, which was divided into aliquots and frozen at −80 °C for further analysis. 

### 2.4. Plasma Inflammatory Cytokines and Adiponectin

Plasma TNF-α, IL-6, IL-8 and IL-10 were measured simultaneously and in duplicate in the same assay using the Human CVD Panel 3 MILLIPLEX^®^ MAP kit following the manufacturer’s instructions (Millipore Corporation, Billerica, MA, USA). Plasma adiponectin was measured in duplicate using the Human CVD Panel 1 MILLIPLEX^®^MAP kit following the protocol of the same manufacturer. These assays are based on Luminex^®^ xMAP^®^ technology (Austin, TX, USA), which uses color-coded microspheres with specific capture antibodies to quantify multiple molecules in the same sample. The results were obtained using the Luminex^®^ IS 200 system, which identifies individual microspheres and quantifies the results based on a secondary antibody/reporter signal. All assays were conducted in the same day to decrease variability. The intra-assay variability was less than 5%.

### 2.5. Plasma LDL Oxidation and Urinary 8-Isoprostanes

Oxidized LDL (oxLDL) was measured by using the monoclonal antibody 4E6 and utilizing the Mercodia oxidized LDL ELISA kit (cat. #10-1143-01). The antibody 4E6 is directed against a conformational epitope in the apoB-100 moiety of LDL, generated as a consequence of substitution of at least 60 lysine residues of apoB-100 with aldehydes [18]. Subjects were asked to collect two 24 h-urine collections at the end of each treatment, respectively, to measure urinary isoprostanes as previously reported [16].

### 2.6. Peripheral Blood Mononuclear Cells (PBMC) Isolation

Isolation of mononuclear cells from whole blood was done following the method used by Boyum *et al.* [19]. Forty milliliters of blood was diluted with 20 mL HBSS (Sigma-Aldrich, St. Louis, MO, USA). The diluted solution was then layered over 20 mL of Histopaque 1077 (Sigma-Aldrich, St. Louis, MO, USA) and centrifuged for 30 min at 500× *g* (Rotanta 460 R, Hettich Lab Technology, Tuttlingen, Germany). The PBMC were removed and washed with HBSS and centrifuged twice at 500× *g* for 10 min. The pellet was re-suspended in 200 μL Tris buffer (10 mmol of Tris, 150 mmol of NaCl and 1 mmol of CaCl_2_ per L, pH 7.4) and divided into two pools for the RNA and nuclear extraction protocols, respectively. The total and relative numbers of PBMC were determined using a hematologic cell counter.

### 2.7. RNA Extraction and Real-Time Polymerase Chain Reaction (PCR)

PBMC in the Tris buffer were centrifuged at 500× *g* for 5 min to obtain a pellet and continue with the method described by Park *et al.* [20]. The pellet was washed twice with cold phosphate buffered saline (PBS) and 1 mL of TRIzol reagent (Invitrogen, Carlsbad, CA, USA) was added to isolate total RNA. One microgram of total RNA was treated with DNase I (Promega, Madison, WI, USA) to remove genomic DNA contamination, and subsequently, RNA samples were reverse transcribed by MMLV reverse transcriptase (Promega, Madison, WI, USA). Real-time PCR analysis was performed using the Sybr Green procedure and a CFX384 real-time PCR detection system (BioRad, Hercules, CA, USA). Primers for superoxide dismutase 1 (SOD1) and 2 (SOD2), glutathione peroxidase 1 (GPx1) and 4 (GPx4), iNOS, NOX2 and the reference gene glyceraldehyde-3-phosphate dehydrogenase (GAPDH) were designed according to GenBank database using Beacon Designer 7 (Premier Biosoft International; Palo Alto, CA, USA). Expression of mRNA values was calculated as previously reported [20].

### 2.8. Statistical Analysis

Data are presented as means ± S.D. The Student’s paired *t*-test was used to compare between periods for all participants. The Student’s non-paired t-test was used to compare MetS characteristics between dyslipidemic and non-dyslipidemic individuals and changes between placebo and GRAPE using SPSS software (version 17.0, SPSS Inc., Chicago, IL, USA). Differences with a *p* ≤ 0.05 were considered significant. 

## 3. Results

### 3.1. MetS Criteria & Study Population

The baseline characteristics of MetS for the participants classified as dyslipidemic or non-dyslipidemic are presented in Table 1. There were no significant differences in age between groups; most of the participants were Caucasian (*n* = 22); there was one Latino in the non-dyslipidemic and one Asian in the dyslipidemic group. By definition, plasma TG were higher (*p* < 0.001) and HDL lower (*p* < 0.01) in individuals with dyslipidemia (Table 1). Interestingly, the non-dyslipidemic subjects had higher plasma glucose (*p* < 0.05), while WC and blood pressure did not differ between groups. The participants’ compliance was greater than 90%, and there were no changes in their diet or physical activity during the whole study [17]. As previously reported, all participants significantly decreased their resting systolic BP (*p* < 0.0025) after the GRAPE period compared with placebo [17]. When participants were analyzed based on the presence of dyslipidemia, we observed a slight but significant decrease in body weight (from 100.9 ± 20.7 to 99.5 ± 13.8 kg) and BMI (from 31.9 ± 5.9 to 31.6 ± 5.6 kg/m^2^) only in those without dyslipidemia after the GRAPE period (*p* < 0.05), while the changes in weight (95.7 ± 13.0 to 95.5 ± 13.8 kg) and BMI (95.7 to 95.5 kg/m^2^) were not significant for the dyslipidemic participants.

**Table 1:** Baseline characteristics of men with metabolic syndrome with dyslipidemia *versus* those without dyslipidemia *.

Parameter	Dyslipidemia ( *n* = 11)	Non-Dyslipidemia ( *n* = 13)	*p* Value
Age	48.1 ± 11.3	53.9 ± 7.4	NS
Waist Circumference (cm)	107.4 ± 10.5	109.2 ± 16.1	NS
Plasma Triglycerides (mg/dL)	222 ± 83 ^a^	146 ± 70 ^b^	*p* < 0.025
Plasma HDL-C (mg/dL)	30 ± 5 ^a^	43 ± 6 ^b^	*p* < 0.001
Plasma Glucose (mg/dL)	99 ± 7 ^a^	107 ± 9 ^b^	*p* < 0.025
Systolic Blood Pressure (mm Hg)	126 ± 13	131 ± 11	NS
Diastolic Blood Pressure (mm Hg)	83 ± 7	85 ± 7	NS

* Dyslipidemia = high TG (≥150 mg/dL) + low HDL (<40 mg/dL); Non-Dyslipidemia = high TG or low HDL or any of the other MetS variables; Values are means ± SD; Numbers with different superscript are significantly different as determined by non-paired Student’s *t*-test.

### 3.2. Plasma Inflammatory Markers and Adiponectin

Changes in adiponectin and in IL-10, as well as changes in inflammatory markers between the GRAPE and placebo periods, are shown in Table 2. Plasma IL-10 (*p* < 0.05) and adiponectin concentrations (*p* < 0.05) followed opposite outcomes based on dyslipidemia category. Changes in plasma IL-6, IL-8, and TNF-α levels did not differ between periods regardless of dyslipidemia classification.

**Table 2:** Change in plasma inflammatory markers between GRAPE and placebo periods for participants classified with dyslipidemia *versus* those without dyslipidemia *.

Change between GRAPE and Placebo	Dyslipidemia ( *n* = 11)	Non-Dyslipidemia ( *n* = 13)	*p* Value
Adiponectin (μg/mL)	−1.749 ± 3.341 ^a^	1.137 ± 3.930 ^b^	*p* < 0.05
IL-10 (pg/mL)	−2.830 ± 3.988 ^a^	1.838 ± 2.738 ^b^	*p* < 0.005
IL-6 (pg/mL)	0.264 ± 1.085	−0.175 ± 1.171	NS
IL-8 (pg/mL)	−0.061 ± 0.534	0.115 ± 0.383	NS
TNF-α (pg/mL)	0.057 ± 0.782	0.175 ± 0.647	NS

* Dyslipidemia = high TG (≥150 mg/dL) + low HDL (<40 mg/dL); Non-Dyslipidemia = high TG or low HDL or any of the MetS variables; Values are means ± SD. Numbers with different superscript are significantly different as determined by non-paired Student’s *t*-test.

### 3.3. Gene Expression of Inflammatory and Oxidative Stress Markers

The changes in the mRNA relative expression of the antioxidant enzymes SOD1, SOD2, GPx1 and GPx4 in the PBMC were similar for both groups after the GRAPE and placebo periods (Table 3). However, there was a significant difference in iNOS mRNA, where an increase was observed in the non-dyslipidemic subjects and a decrease in those individuals with dyslipidemia.

**Table 3:** Changes in mRNA expression of proteins associated with oxidative stress in peripheral blood mononuclear cells between GRAPE and placebo periods for participants classified with dyslipidemia *versus* those without dyslipidemia *.

Change between GRAPE and Placebo	Dyslipidemia ( *n* = 11)	Non-Dyslipidemia ( *n* = 13)	*p* Value
NOX2 (arbitrary units)	0.179 ± 0.514	−0.004 ± 0.726	NS
SOD1 (arbitrary units)	0.281 ± 1.335	−0.355 ± 1.289	NS
SOD2 (arbitrary units)	0.927 ± 2.917	−0.165 ± 1.383	NS
GPX1 (arbitrary units)	0.460 ± 1.793	−0.152 ± 0.722	NS
GPX4 (arbitrary units)	0.196 ± 0.738	−0.196 ± 0.798	NS
iNOS (arbitrary units)	−0.460 ± 0.913 ^a^	0.449 ± 0.726 ^b^	*p* < 0.025

* Dyslipidemia = high TG (≥150 mg/dL) + low HDL (<40 mg/dL); Non-Dyslipidemia = high TG or low HDL or any of the MetS variables; Values are expressed as mean ± SD for the number of participants indicated in parentheses. Numbers with different superscript are significantly different as determined by non-paired Student’s *t*-test.

### 3.4. Oxidative Stress Markers

Changes in plasma oxLDL and in urinary 8-isoprostanes were not different between the GRAPE and placebo periods regardless of dyslipidemia classification (data not shown).

**Figure 1:**
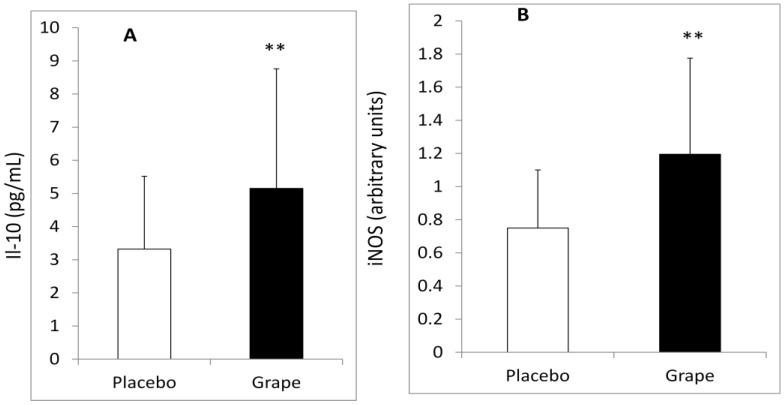
(**A**) Concentrations of plasma IL-10 and (**B**) Relative gene expression of iNOS in PBMC of non-dyslipidemic individuals (*n* = 13) during the GRAPE (black bar) and the placebo (white bar) periods. Values are mean ± SD. ** Indicates significantly different from placebo at *p* < 0.001.

In addition, plasma IL-10 levels were higher in the GRAPE period compared with placebo in participants without dyslipidemia (*p* < 0.005, Figure 1A). 

Notably, we did observe that the changes in iNOS mRNA expression in the PBMC after the GRAPE period, compared with placebo, were significantly higher in individuals without dyslipidemia (Figure 1B).

### 3.5. Correlations between Plasma and Gene Expression Markers

**Figure 2:**
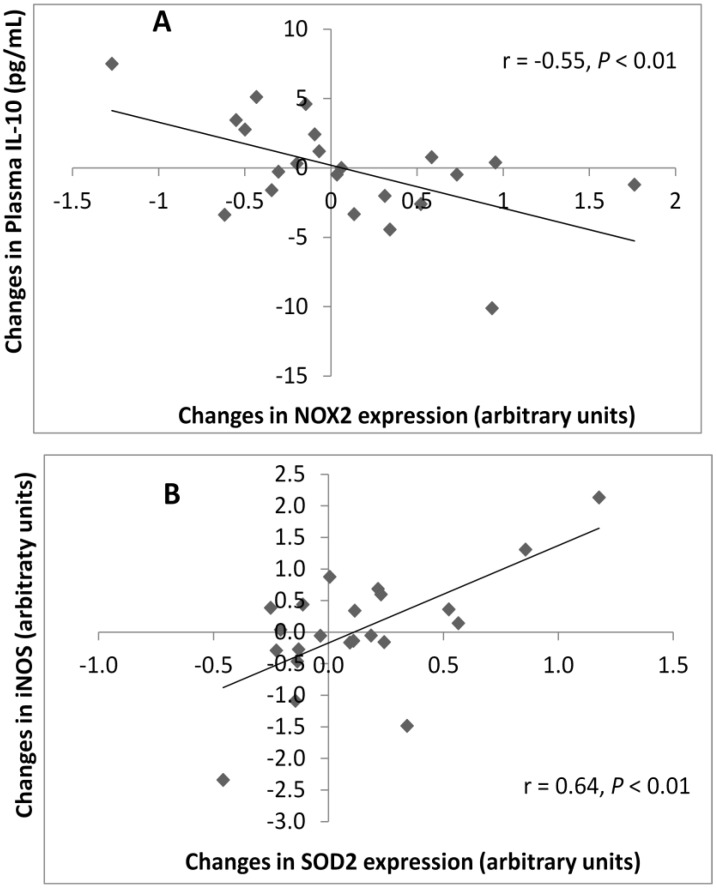
(**A**) Correlations between changes in plasma IL-10 concentrations and NOX2 relative gene expression (*r* = −0.55, *p* < 0.01); (**B**) Correlations between the changes in the relative gene expression of iNOS and changes in SOD2 (*r* = 0.64, *p* < 0.001).

Changes in plasma IL-10 were negatively correlated with NOX2 expression (*r* = −0.55, *p* < 0.01, Figure 2A), and changes in iNOS expression were positively correlated with SOD2 (*r* = 0.642, *p* < 0.01, Figure 2B). 

## 4. Discussion

In this study, we have demonstrated that grape consumption increases IL-10 and adiponectin, two anti-inflammatory cytokines only in those subjects who do not present dyslipidemia (elevated TG and low HDL-C). Further, the changes in iNOS expression between the GRAPE and placebo periods were significantly different when we compared the dyslipidemic *versus* the non-dyslipidemic subjects. We believe these beneficial effects on inflammation are attributed to the polyphenols present in the GRAPE. 

Overproduction of TG-rich large VLDL, which is a feature of dyslipidemia in MetS [21], initiates a sequence of plasma lipoprotein changes, resulting in TG-rich LDL and HDL by the action of cholesteryl ester transfer protein [21,22]. These particles are preferred substrates for hepatic lipase, resulting in further formation of smaller and denser LDL and HDL [21,22]. The small, dense HDL is more rapidly cleared from plasma, while the small, dense LDL can penetrate the arterial wall more rapidly and become oxidized, which promotes foam cell formation and production of inflammatory molecules [22]. In addition, lipolysis of TGRL and/or their remnants have been shown to induce inflammation and foam cell formation, similar to LDL [8]. Thus, people with MetS having dyslipidemia with lower anti-inflammatory HDL and a higher number of pro-inflammatory small dense LDL and TGRL remnants may have a more inflammatory environment, rendering their cells less responsive than people with MetS without dyslipidemia to dietary interventions, as this study suggests. 

Circulating IL-10 is mainly derived from T-cells, B-cells, monocytes and macrophages [23]. About 75% of the variance in IL-10 production capacity in humans is under genetic control [11]. Lower levels of serum IL-10 have been significantly associated with MetS, independent of age and body weight [10]. Low production capacity of IL-10, using a whole-blood assay and lipopolysaccharide as stimulus, has also been reported in this population [11]. IL-10 acts in a feedback loop to inhibit continued pro-inflammatory cytokine production by macrophages or lymphocytes [23]. It has been demonstrated that pro-inflammatory cytokines (e.g., TNF-α) induce hypertriglyceridemia [9]. Thus, it is possible that our dyslipidemic individuals may have low innate IL-10 production capacity, which fails to limit the chronic pro-inflammatory state characteristic of MetS, with the subsequent observed dyslipidemic effects. Moreover, it was demonstrated that non-heritable factors, such as life-style changes, may have a role in the regulation of IL-10 levels, but only in those capable of increasing their IL-10 production capacity [10]. Thus, we observed that only participants without dyslipidemia (possibly with normal IL-10 production capacity) responded favorably to GRAPE consumption by increasing their plasma IL-10 levels.

Secretion of adiponectin, an important anti-inflammatory and insulin sensitizing adipokine, is reduced in obesity, insulin resistance and type 2 diabetes and increased by weight loss [5]. It was shown that chronic consumption (12 weeks) of a polyphenolic grape seed extract in hamsters fed a high-fat diet prevented body weight gain and increased adiponectin levels by 61% compared with high-fat fed obese controls [24]. Although animal and human responses are not always comparable, we also found that after grape consumption participants increased their plasma levels of adiponectin, compared to placebo; however, this effect was only observed in individuals without dyslipidemia. We also observed in these same participants a slight, but significant decrease in their body weight and BMI after GRAPE compared to placebo. At this point, it is not clear whether the observed increase in adiponectin levels could be directly related to the effects of GRAPE consumption on adipocytes or indirectly as a result of reductions in body weight. It is possible that the clinical contribution of this small, but significant, reduction in body weight of our participants could have modulated adiponectin levels in this study.

It has been shown that adiponectin induces expression of IL-10 in human macrophages and partially mediates the anti-atherogenic effects of adiponectin [25]. Because serum IL-10 is positively associated with adiponectin mainly in MetS individuals [26], the increases in adiponectin after GRAPE consumption might have induced production of IL-10 in our participants. In addition, we did find that changes in plasma IL-10 were negatively correlated with NOX2 expression in PBMC, potentially reflecting the anti-inflammatory actions of IL-10, as has been reported [23]. 

Numerous studies with polyphenols [14,27] have shown reductions in iNOS expression using macrophages and other cells under inflammatory conditions [27] or in atherosclerotic plaques [28] considered to be an anti-inflammatory effect of polyphenols [27]. However, it was reported that dealcoholized red wine polyphenols induced NO production and mRNA expression of iNOS in healthy human PBMC [29]. The authors suggested mononuclear cells could represent an additional source of NO (besides endothelial-derived NO), contributing to an increase in plasma NO levels under the effects of polyphenols [29]. In agreement, we found that iNOS expression in the PBMC isolated from our participants was significantly higher after they consumed GRAPE, compared with placebo, but only in subjects without dyslipidemia.

The antioxidant enzymes SODs play a critical role in inhibiting oxidative inactivation of NO, enhancing its bioavailability [30]. We found that iNOS expression changes were positively correlated with the antioxidant enzyme SOD2, suggesting that NO produced by iNOS of PBMCs would be protected from oxidative inactivation. Thus, PBMC-derived NO may then retain bioactivity and potentially contribute to plasma NO pool.

Different from this study, other investigations have shown positive effects of grape derivatives in oxidative stress and antioxidant markers. A previous and similar study [16] from our lab showed that lyophilized grape powder decreased urinary 8-isoprostanes, a marker of oxidative stress, and the pro-inflammatory cytokine TNF-α in otherwise healthy pre- and post-menopausal women. We studied the effects of GRAPE on these same markers; however, by definition, our population presented established CVD risk factors that may have required more time of supplementation. Other studies showed that red grape juice polyphenols may reduce oxidative stress by decreasing *ex vivo* neutrophil NOX activity in hemodialysis patients [31] and both mRNA and protein levels of NOX2 in human neutrophils and mononuclear blood cells incubated with this grape juice [32]. Similarly, an *in vitro* study showed that grape seed polyphenols induced endogenous antioxidant enzymes in cardiac cells [33]. 

There are some limitations to this study, one being that we did not measure plasma polyphenols to determine whether half-life was shorter in dyslipidemic individuals. All blood draws were taken after 12 h of fasting to have accurate values for plasma lipids. It is well known that the half-life of polyphenols is very short, between 2 and 4 h [34], thus, under these circumstances, we could not evaluate differences in polyphenol content in plasma. 

## 5. Conclusions

In conclusion, we found that participants without dyslipidemia showed a more favorable response to GRAPE consumption by increasing plasma IL-10 and adiponectin levels, compared to placebo. In addition, iNOS expression in PBMC from non-dyslipidemic individuals was higher after consuming GRAPE. It may be possible that in this specific population of MetS subjects classified with dyslipidemia, their cells are exposed to a more inflammatory environment, which we could not detect, thus, rendering their cells less responsive to the dietary intervention implemented in this study. In addition, it could also be possible that the grape phenolics are oxidized at a higher rate in dyslipidemia, and, therefore, not available to modulate other anti-inflammatory pathways in this specific population with MetS.

## References

[1] Alberti K.G., Eckel R.H., Grundy S.M., Zimmet P.Z., Cleeman J.I., Donato K.A., Fruchart J.C., James W.P., Loria C.M., Smith S.C. (2009). Harmonizing the metabolic syndrome: A joint interim statement of the International Diabetes Federation Task Force on Epidemiology and Prevention; National Heart, Lung, and Blood Institute; American Heart Association; World Heart Federation; International Atherosclerosis Society; and International Association for the Study of Obesity. Circulation.

[2] Grundy S.M., Brewer H.B., Cleeman J.I., Smith S.C., Lenfant C. (2004). Definition of metabolic syndrome: Report of the National Heart, Lung, and Blood Institute/American Heart Association conference on scientific issues related to definition. Circulation.

[3] Furukawa S., Fujita T., Shimabukuro M., Iwaki M., Yamada Y., Nakajima Y., Nakayama O., Makishima M., Matsuda M., Shimomura I. (2004). Increased oxidative stress in obesity and its impact on metabolic syndrome. J. Clin. Invest..

[4] Devaraj S., Rosenson R.S., Jialal I. (2004). Metabolic syndrome: An appraisal of the pro-inflammatory and procoagulant status. Endocrinol. Metab. Clin. North Am..

[5] Gustafson B., Hammarstedt A., Andersson C.X., Smith U. (2007). Inflamed adipose tissue: A culprit underlying the metabolic syndrome and atherosclerosis. Arterioscler. Thromb. Vasc. Biol..

[6] De Matthaeis A., Greco A., Serviddio G., Stramaglia G., Vendemiale G. (2010). Endothelial dysfunction evaluated by flow mediated dilation is strongly associated to metabolic syndrome in the elderly. Aging Clin. Exp. Res..

[7] Choi K.M., Ryu O.H., Lee K.W., Kim H.Y., Seo J.A., Kim S.G., Kim N.H., Choi D.S., Baik S.H. (2007). Serum adiponectin, interleukin-10 levels and inflammatory markers in the metabolic syndrome. Diabetes Res. Clin. Pract..

[8] Schwartz E.A., Reaven P.D. (2012). Lipolysis of triglyceride-rich lipoproteins, vascular inflammation, and atherosclerosis. Biochim. Biophys. Acta.

[9] Feingold K.R., Grunfeld C. (1992). Role of cytokines in inducing hyperlipidemia. Diabetes.

[10] Esposito K., Pontillo A., Giugliano F., Giugliano G., Marfella R., Nicoletti G., Giugliano D. (2003). Association of low interleukin-10 levels with the metabolic syndrome in obese women. J. Clin. Endocrinol. Metab..

[11] Van Exel E., Gussekloo J., de Craen A.J., Frolich M., Bootsma-van der Wiel A., Westendorp R.G. (2002). Low production capacity of interleukin-10 associates with the metabolic syndrome and type 2 diabetes: The Leiden 85-Plus Study. Diabetes.

[12] Zern T.L., Fernandez M.L. (2005). Cardioprotective effects of dietary polyphenols. J. Nutr..

[13] Magrone T., Jirillo E. (2010). Polyphenols from red wine are potent modulators of innate and adaptive immune responsiveness. Proc. Nutr. Soc..

[14] Santangelo C., Vari R., Scazzocchio B., di Benedetto R., Filesi C., Masella R. (2007). Polyphenols, intracellular signalling and inflammation. Ann. Ist. Super. Sanita.

[15] Xia E.Q., Deng G.F., Guo Y.J., Li H.B. (2010). Biological activities of polyphenols from grapes. Int. J. Mol. Sci..

[16] Zern T.L., Wood R.J., Greene C., West K.L., Liu Y., Aggarwal D., Shachter N.S., Fernandez M.L. (2005). Grape polyphenols exert a cardioprotective effect in pre- and postmenopausal women by lowering plasma lipids and reducing oxidative stress. J. Nutr..

[17] Barona J., Aristizabal J.C., Blesso C.N., Volek J.S., Fernandez M.L. (2012). Grape polyphenols reduce blood pressure and increase flow-mediated vasodilation in men with metabolic syndrome. J. Nutr..

[18] Holvoet P., Macy E., Landeloos M., Jones D., Jenny N.S., van de Werf F., Tracy R.P. (2006). Analytical performance and diagnostic accuracy of immunometric assays for the measurement of circulating oxidized LDL. Clin. Chem..

[19] Böyum A. (1968). Isolation of mononuclear cells and granulocytes from human blood. Isolation of monuclear cells by one centrifugation, and of granulocytes by combining centrifugation and sedimentation at 1 g. Scand. J. Clin. Lab. Invest. Suppl..

[20] Park Y.K., Rasmussen H.E., Ehlers S.J., Blobaum K.R., Lu F., Schlegal V.L., Carr T.P., Lee J.Y. (2008). Repression of proinflammatory gene expression by lipid extract of Nostoc commune var sphaeroides Kutzing, a blue-green alga, via inhibition of nuclear factor-kappaB in RAW 26.7 macrophages. Nutr. Res..

[21] Adiels M., Olofsson S.O., Taskinen M.R., Boren J. (2008). Overproduction of very low-density lipoproteins is the hallmark of the dyslipidemia in the metabolic syndrome. Arterioscler. Thromb. Vasc. Biol..

[22] Raal F.J. (2009). Pathogenesis and management of the dyslipidemia of the metabolic syndrome. Metab. Syndr. Relat. Disord..

[23] Tedgui A., Mallat Z. (2001). Anti-inflammatory mechanisms in the vascular wall. Circ. Res..

[24] Decorde K., Teissedre P.L., Sutra T., Ventura E., Cristol J.P., Rouanet J.M. (2009). Chardonnay grape seed procyanidin extract supplementation prevents high-fat diet-induced obesity in hamsters by improving adipokine imbalance and oxidative stress markers. Mol. Nutr. Food Res..

[25] Kumada M., Kihara S., Ouchi N., Kobayashi H., Okamoto Y., Ohashi K., Maeda K., Nagaretani H., Kishida K., Maeda N. (2004). Adiponectin specifically increased tissue inhibitor of metalloproteinase-1 through interleukin-10 expression in human macrophages. Circulation.

[26] Nishida M., Moriyama T., Sugita Y., Yamauchi-Takihara K. (2007). Interleukin-10 associates with adiponectin predominantly in subjects with metabolic syndrome. Circ. J..

[27] Tunon M.J., Garcia-Mediavilla M.V., Sanchez-Campos S., Gonzalez-Gallego J. (2009). Potential of flavonoids as anti-inflammatory agents: Modulation of pro-inflammatory gene expression and signal transduction pathways. Curr. Drug Metab..

[28] Mallat Z., Heymes C., Ohan J., Faggin E., Leseche G., Tedgui A. (1999). Expression of interleukin-10 in advanced human atherosclerotic plaques: Relation to inducible nitric oxide synthase expression and cell death. Arterioscler. Thromb. Vasc. Biol..

[29] Magrone T., Tafaro A., Jirillo F., Panaro M.A., Cuzzuol P., Cuzzuol A.C., Pugliese V., Amati L., Jirillo E., Covelli V. (2007). Red wine consumption and prevention of atherosclerosis: An *in vitro* model using human peripheral blood mononuclear cells. Curr. Pharm. Des..

[30] Fukai T., Ushio-Fukai M. (2011). Superoxide dismutases: Role in redox signaling, vascular function, and diseases. Antioxid. Redox Signal..

[31] Castilla P., Davalos A., Teruel J.L., Cerrato F., Fernandez-Lucas M., Merino J.L., Sanchez-Martin C.C., Ortuno J., Lasuncion M.A. (2008). Comparative effects of dietary supplementation with red grape juice and vitamin E on production of superoxide by circulating neutrophil NADPH oxidase in hemodialysis patients. Am. J. Clin. Nutr..

[32] Davalos A., de la Pena G., Sanchez-Martin C.C., Teresa Guerra M., Bartolome B., Lasuncion M.A. (2009). Effects of red grape juice polyphenols in NADPH oxidase subunit expression in human neutrophils and mononuclear blood cells. Br. J. Nutr..

[33] Du Y., Guo H., Lou H. (2007). Grape seed polyphenols protect cardiac cells from apoptosis via induction of endogenous antioxidant enzymes. J. Agric. Food Chem..

[34] Borges G., Mullen W., Mullan A., Lean M.E., Roberts S.A., Crozier A. (2010). Bioavailability of multiple components following acute ingestion of a polyphenol-rich juice drink. Mol. Nutr. Food Res..

